# Comparison of extended reality and conventional methods of basic life support training: protocol for a multinational, pragmatic, noninferiority, randomised clinical trial (XR BLS trial)

**DOI:** 10.1186/s13063-021-05908-z

**Published:** 2021-12-20

**Authors:** Dong Keon Lee, Chang Woo Im, You Hwan Jo, Todd Chang, Joo Lee Song, Cindy Luu, Ralph Mackinnon, Suresh Pillai, Chuen Neng Lee, Sanghoon Jheon, Soyeon Ahn, Seung Hyun Won

**Affiliations:** 1 Department of Emergency Medicine, Seoul National University Bundang Hospital, 13620, 82, Gumi-ro 173beon-gil, Bundang-gu, Seongnam, Gyeonggi-do Republic of Korea; 2 Department of Emergency Medicine, Seoul National University College of Medicine, Seoul, Republic of Korea; 3 Division of Emergency and Transport Medicine, Department of Pediatrics, Children’s Hospital Los Angeles, Keck School of Medicine, University of Southern California, Los Angeles, USA; 4 Department of Anaesthesia, Royal Manchester Children’s Hospital, Manchester, UK; 5 Centre for Healthcare Simulation, Yong Loo Lin School of Medicine, National University of Singapore, Singapore, Singapore; 6 Department of Surgery, Yong Loo Lin School of Medicine, National University of Singapore, Singapore, Singapore; 7 Department of Thoracic and Cardiovascular Surgery, Seoul National University Bundang Hospital, Seoul National University College of Medicine, Seoul, Republic of Korea; 8 Division of Statistics, Medical Research Collaborating Centre, Seoul National University Bundang Hospital, Seongnam, Republic of Korea

**Keywords:** Cardiopulmonary resuscitation, Basic life support, Virtual reality, Extended reality, Education

## Abstract

**Background:**

Conventional cardiopulmonary resuscitation (CPR) training for the general public involves the use of a manikin and a training video, which has limitations related to a lack of realism and immersion. To overcome these limitations, virtual reality and extended reality technologies are being used in the field of medical education. The aim of this study is to explore the efficacy and safety of extended reality (XR)-based basic life support (BLS) training.

**Methods:**

This study is a prospective, multinational, multicentre, randomised controlled study. Four institutions in 4 countries will participate in the study. A total of 154 participants will be randomly assigned to either the XR group or the conventional group stratified by institution and sex (1:1 ratio). Each participant who is allocated to either group will be sent to a separate room to receive training with an XR BLS module or conventional CPR training video. All participants will perform a test on a CPR manikin after the training. The primary outcome will be mean compression depth. The secondary outcome will be overall BLS performance, including compression rate, correct hand position, compression, and full release and hands-off time.

**Discussion:**

Using virtual reality (VR) to establish a virtual educational environment can give trainees a sense of realism. In the XR environment, which combines the virtual world with the real world, trainees can more effectively learn various skills. This trial will provide evidence of the usefulness of XR in CPR education.

**Trial registration:**

ClinicalTrials.gov NCT04736888. Registered on 29 January 2021

**Supplementary Information:**

The online version contains supplementary material available at 10.1186/s13063-021-05908-z.

## Introduction

### Background and rationale

In Europe and the USA, more than 300,000 cases of out-of-hospital cardiac arrest (OHCA) occur annually, and OHCA is a significant global health problem [1, 2]. To improve the outcomes of patients with OHCA, the early recognition of cardiac arrest and performance of cardiopulmonary resuscitation (CPR) by bystanders are the most essential steps. However, bystander CPR is provided to patients with OHCA in less than 50% of cases [3, 4]. Therefore, it is very important to provide CPR training to the general public.

Conventional CPR training consists of instructor-led in-person training after an online course. Although several feedback devices have been developed to improve the quality of the training, they were neither realistic nor immersive [5]. In addition, in conventional training programmes, trainees are constrained in terms of time and location, as they are usually kept to a schedule.

Virtual reality (VR) technology designed to maximise immersion has been shown to be able to overcome these limitation s[6]. Nas et al. reported a comparable chest compression rate but inferior compression depth with VR CPR training compared to conventional face-to-face training [7]. These results suggest that although VR has advantages over conventional education methods, it is difficult to perform techniques such as chest compression, ventilation, and defibrillation with real senses [8].

On the other hand, extended reality (XR), which combines the virtual and real worlds, could alleviate these shortcomings by using real manikins in a virtual environment, and consequently, it could be used to conduct realistic and immersive training without being constrained by space and time.

### Objectives

The aim of this study is to compare the efficacy of the XR-based basic life support (BLS) training method, which combines VR and the use of manikins, with that of the conventional training method. The hypothesis of this study is that the BLS skills gained by the XR method are not inferior to those gained by the conventional method.

### Trial design

**Fig. 1:**
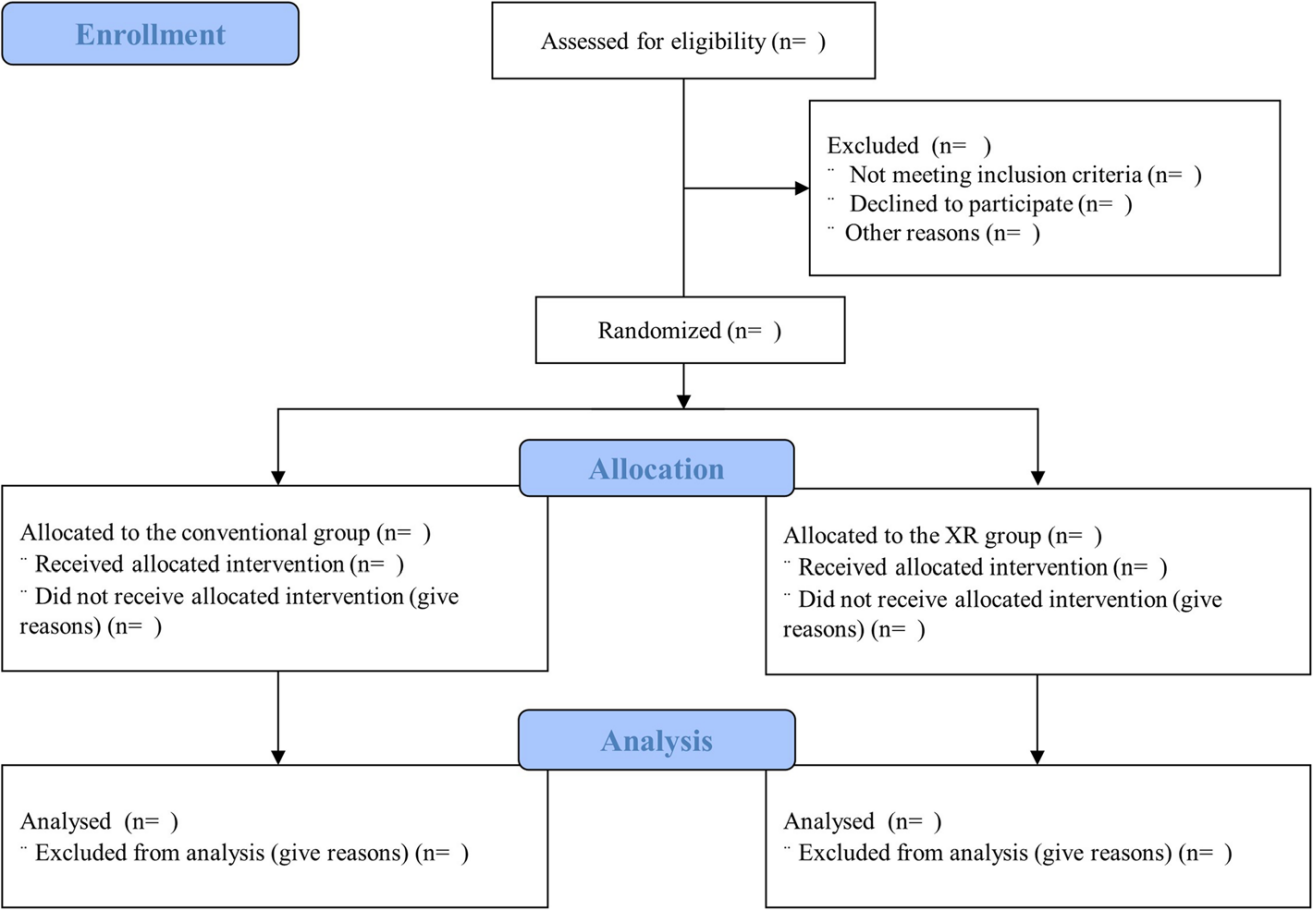
Study algorithm. Q&A question and answer

**Fig. 2:**
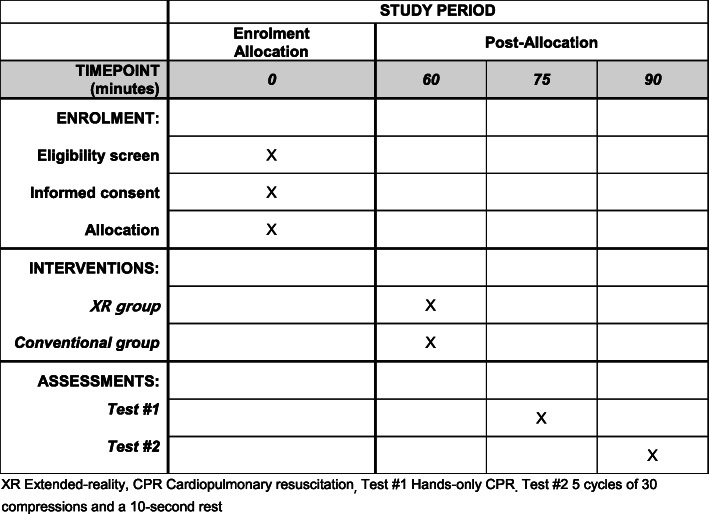
Schedule of enrolment, interventions, and assessments according to the Standard Protocol Items: Recommendations for Interventional Trials (SPIRIT) guideline. XR, extended reality; CPR, cardiopulmonary resuscitation; Test #1, hands-only CPR; Test #2, 5 cycles of 30 compressions and a 10-s rest

This study is a prospective, multinational, pragmatic, noninferiority, randomised controlled study. Four institutions in 4 countries will participate in the study. The trial protocol will be approved by the Institutional Review Boards (IRBs) of the individual participating institutions (Fig. 1). This study was registered at ClinicalTrials.gov (National Clinical Trial (NCT) number: NCT04736888), and the protocol was designed following the guidelines of the Consolidated Standards of Reporting Trials and Standard Protocol Items: Recommendations for Interventional Trials (SPIRIT) (Supplementary file 1 SPIRIT 2013 checklist) (Fig. 2).

## Methods: participants, interventions, and outcomes

### Study setting

We designed a multinational, multicentre, randomised clinical trial that will include 154 participants from Seoul National University Bundang Hospital (South Korea), Children’s Hospital Los Angeles (USA), Royal Manchester Children’s Hospital (UK), and National University of Singapore (Singapore).

CBS 2.0™ (Tetra Signum, Inc., Seoul, South Korea), a self-learning type of XR solution, will be used for XR-based BLS training. A total of 154 participants will be enrolled after recruitment is announced, and a stratified permuted block will be used for randomisation. We plan to begin trial recruitment in March 2021, and the planned study period will be 6 months.

### Eligibility criteria

Individuals who are not healthcare providers and are 18 years old or older will be eligible for inclusion. Participants who are not capable of performing either the training or the CPR test due to physical or cognitive limitations will be excluded [7, 9–11]. Those who have upper extremity injuries or are pregnant will also be excluded [12]. If participants experience dizziness, headache, or motion sickness during the 2-min XR device adaptation period that prevents them from participating in the simulation study, they will also be excluded [13].

### Ethics approval

The investigators of each institution will explain to the participants the purpose of the study, the risks and benefits of the research, and how to contact the study investigators (or the Seoul National University Bundang Hospital ethics review board) regarding the problems that occurred in this study prior to obtaining their informed consent (Supplementary file 4 INFORMATION AND INFORMED CONSENT FORM).

## Interventions

### Explanation of the choice of comparators

Participants will be randomly assigned to (1) the XR group or (2) the conventional group stratified by institution and sex. Because an instructor-led in-person training course after an online course is the current standard CPR training method, the conventional group has been chosen as the control group.

### Intervention description

Participants will be randomly assigned to either the XR group or the conventional group (1:1 ratio). Each participant who is allocated to either group will be sent to a separate room (room A for the XR group and room B for the conventional group) and trained with an XR BLS module or conventional CPR training video.

Conventional CPR training consists of instructor-led in-person training with a BLS video and a manikin equipped with a feedback device. XR group participants, on the other hand, are trained through self-training using the XR BLS module without an instructor.

**Fig. 3:**
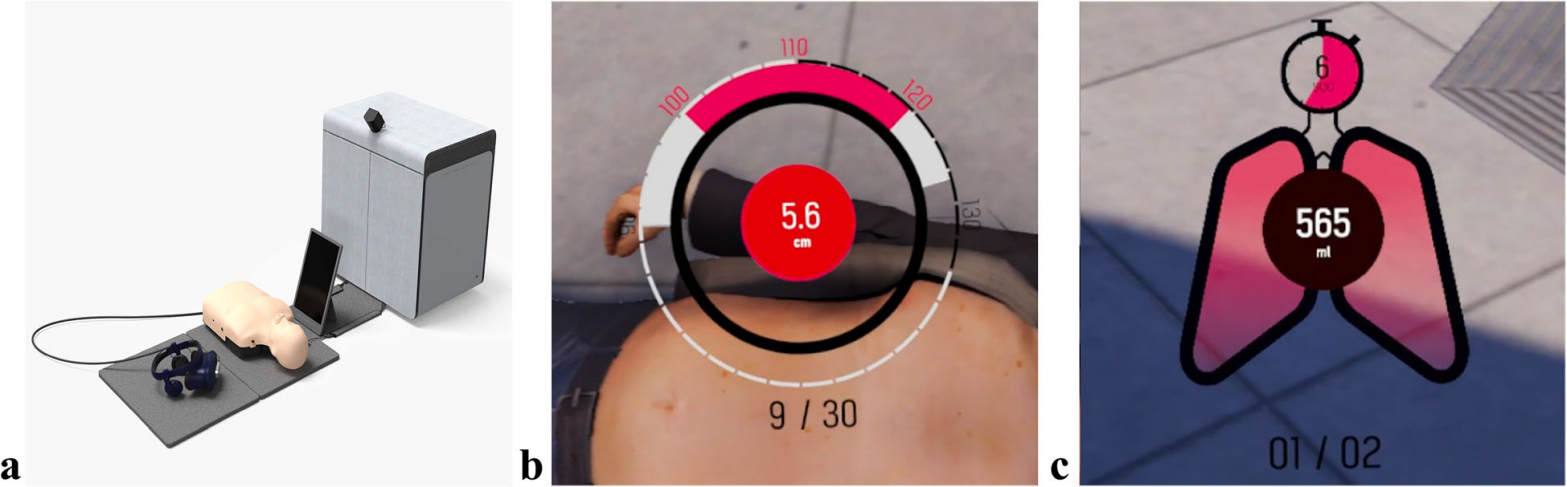
Extended reality basic life support module. **a** Components of the extended reality basic life support module. **b** The real-time chest compression feedback in virtual reality environment. **c** The real-time ventilation feedback in virtual reality environment

The XR BLS module features a housing unit with an attached base station, a touch screen that students can use to play content, a half-torso manikin (BestCPR, Gimpo, Korea), and an HTC Vive Pro Head-Mounted Display (HMD) with an attached hand tracking device (Leap Motion, UltraLeap, CA, USA) (Fig. 3).

The VR-BLS training application was developed according to the 2020 AHA (American Heart Association) guidelines for BLS [7]. The learning mode consists of two parts: an introductory video explaining the CPR steps and a VR instructor explaining the steps in much more detail. Participants can perform techniques such as chest compression and ventilation on the mannequin in front of them even in a VR environment. The practice mode takes place in a street environment and has a VR instructor programmed to instruct participants to perform each step of CPR (Supplementary video). To adapt to the XR equipment, XR group participants are allotted an additional 2 min.

In both groups, BLS training will take approximately 60 min and is composed of checking responsiveness, calling for help, checking breathing, performing chest compressions, performing rescue breathing, and using a defibrillator. All training in both groups will be conducted by only one participant at a time.

Each group will be trained to perform both standard 30:2 CPR and hands-only CPR. Participants will be taught to use a face mask or cloth to cover the patient’s face and to perform hands-only CPR to reduce the risk of disease transmission when the patient is a suspected or confirmed or unknown case of COVID-19 [14, 15]. In addition, participants will also be taught to perform standard 30:2 CPR. The XR group will follow the instructions of the XR content, while the conventional group will watch the BLS video and follow its instructions [16]. Both groups will have a 5-min Q&A (question and answer) session after training (Fig. 1).

All participants and BLS instructors will wear a mask and follow social distancing guidelines to prevent the transmission of respiratory diseases during the study. There is no other specific relevant concomitant care and interventions that are permitted or prohibited during the trial.

### Test procedure

All participants will take a test at least 10 min after completing the training [10, 11]. The test will be conducted by a BLS instructor who has not been involved in BLS training. The Laerdal Resusci Anne™ manikin (Laerdal Medical Corporation, Stavanger, Norway) will be used. Additionally, depending on the policies of each participating institution, the test will be recorded on a smartphone, video camera, or other recording devices after obtaining consent from the participant.

The test will consist of two parts. Each part will assess the CPR steps of ‘checking for a response’, ‘calling for help’, ‘checking for breathing’, ‘performing chest compressions’, and ‘using an automated electrical defibrillator (AED)’. This study’s main test, test #1, will use hands-only CPR as the primary assessment criteria and will be conducted in line with the American Heart Association (AHA) and European Resuscitation Council (ERC)’s ‘Basic life support guidelines for adults with suspected or confirmed COVID-19’.

Test #2 will assume a scenario in which the patient is not suspected of having COVID-19 and will assess BLS skills with the 30:2 chest compression to rescue breath ratio. However, as face shields and pocket masks do not guarantee protection from the transmission of COVID-19 between the participants via a manikin, the rescue breaths will be substituted by a 10-s rest. To reduce interference between the two tests, after the continuous chest compression assessment (test #1), the assessment of 5 cycles of 30 chest compressions with a 10-s rest between each cycle as a substitute for rescue breaths (test #2) will be conducted after a 10-min break. To evaluate the effectiveness of BLS training, a Laerdal Resusci Anne™ manikin connected to the Laerdal SimPad SkillReporter™ will be used. All records stored on the Skillreporter™ will be analysed on a computer equipped with the Laerdal PC Skill Reporting System (Laerdal Medical Corporation). After the test, all participants will be asked to fill out a questionnaire.

### Criteria for discontinuing or modifying the allocated interventions

During the study, those who experience dizziness, headache, motion sickness, or physical injuries that prevent their participation in this study will be excluded [13]. Participants may choose to discontinue their participation at any time for any reason.

### Provisions for post-trial care

This study investigates the efficacy and safety of the XR-based BLS training method. The probability of side effects during CPR training is very low. If any side effects occur during BLS training, appropriate treatment will be provided by each institution. In the case of serious side effects related to this study, the research director will follow each institution’s IRB protocol for reporting and subsequent measures required.

### Outcomes

#### Primary outcomes

The primary outcome will be the mean compression depth (mm) over 2 min, which will be measured in test #1, during which continuous chest compression will be delivered.

#### Secondary outcomes

The secondary outcomes will be as follows: the total number of chest compressions (*n*), the mean chest compression depth of each 30-s epoch, correct hand position (%), adequate compression depth (%), compression and full release (%), mean compression rate (number per minute), adequate compression rate (%), adequate compression depth and rate (%), and hands-off time (s).

In addition, the successful implementation of the steps involving checking for a response, calling for help, and checking for breathing will be compared. Other test results include the time interval from arrival on the scene (patient contact in the simulation) to the first chest compression (s), the total number of chest compressions with full release, correct hand position (total number of times), and correct AED use. For institutions where the policies allow recordings, the time interval from arrival on the scene (patient contact in the simulation) to the first chest compression (s), time from powering on the AED to defibrillation (s), and the time from checking for a response to defibrillation (s) will be assessed by reviewing the recordings (Tables 1 and 2). Regarding the questionnaire (Supplementary files #2, 3), a 5-point Likert scale will be used to assess the effectiveness of training and self-confidence in CPR after training (1 = strongly disagree, 5 = strongly agree) [17–19]. The demographics of the participants, such as age, sex, weight, height, and status of previous CPR training, will also be investigated (Table 3).

**Table 1:** Test 1 results

Patients checking and calling for help						Valid value
Checking for response						Done/not done
Calling for help						Done/not done
Checking for breathing						Done/not done
**Chest compression**	Total	1st epoch	2nd epoch	3rd epoch	4th epoch	Valid value
*The time interval from arrival on the scene to the first chest compression (s)						0–60
Number of chest compressions (*n*)						0–400
Mean compression depth (mm)						1–100
Number of adequate compression depth (*n*)						0–400
Adequate compression depth (%)						0–100
Mean compression rate (*n*/min)						1–200
Adequate compression rate (%)						0–100
Adequate compression depth and rate (%)						0–100
Number of compressions with correct hand position (*n*)						0–400
Correct hand position (%)						0–100
Number of chest compressions with full release (*n*)						0–400
Compression and full release (%)						0–100
Hands-off time (s)						0–120
**AED**						Valid value
AED use						Done/not done
Correct AED use						Yes/no
*Time from powering on the AED to defibrillation (s)						0–120
*Time from checking for a response to defibrillation (s)						0–300

Epoch, 30-s epochs in test 1

Adequate compression depth, 5–6 cm; adequate compression rate, 100–120/min; correct hand position, hand position as the lower half of the sternum in the centre of the chest; compression and full release, less than or equal to 5-mm residual displacement; hands-off time, interruptions of chest compressions during cardiopulmonary resuscitation

*These variables can be obtained from recording devices such as smartphone cameras and video cameras

**Table 2:** Test 2 results

Patients checking and calling for help						Valid value
Checking for response						Done/not done
Calling for help						Done/not done
Checking for breathing						Done/not done
Chest compression	1st cycle	2nd cycle	3rd cycle	4th cycle	5th cycle	Valid value
*The time interval from arrival on the scene to the first chest compression (s)						0–60
Number of chest compressions (*n*)						0–400
Mean compression depth (mm)						1–100
Number of adequate compression depth (*n*)						0–400
Adequate compression depth (%)						0–100
Mean compression rate (*n*/min)						1–200
Adequate compression rate (%)						0–100
Adequate compression depth and rate (%)						0–100
Number of compressions with correct hand position (*n*)						0–400
Correct hand position (%)						0–100
Number of chest compressions with full release (*n*)						0–400
Compression and full release (%)						0–100
Hands-off time (s)						0–120
AED						Valid value
AED use						Done/not done
Correct AED use						Yes/no
*Time from powering on the AED to defibrillation (s)						0–120
*Time from checking for a response to defibrillation (s)						0–300

Adequate compression depth, 5–6 cm; adequate compression rate, 100–120/min; correct hand position, hand position as the lower half of the sternum in the centre of the chest; compression and full release, less than or equal to 5-mm residual displacement; hands-off time, interruptions of chest compressions during cardiopulmonary resuscitation

*These variables can be obtained from recording devices such as smartphone cameras and video cameras

**Table 3:** Demographics of participants

		Valid value
Age (years)		18–100
Sex		Male, female
Weight (kg)		40–150
Height (cm)		100–200
Previous CPR training		Yes/no

Previous CPR training: participants who have previously taken a CPR education

### Participant timeline

**Fig. 4:**
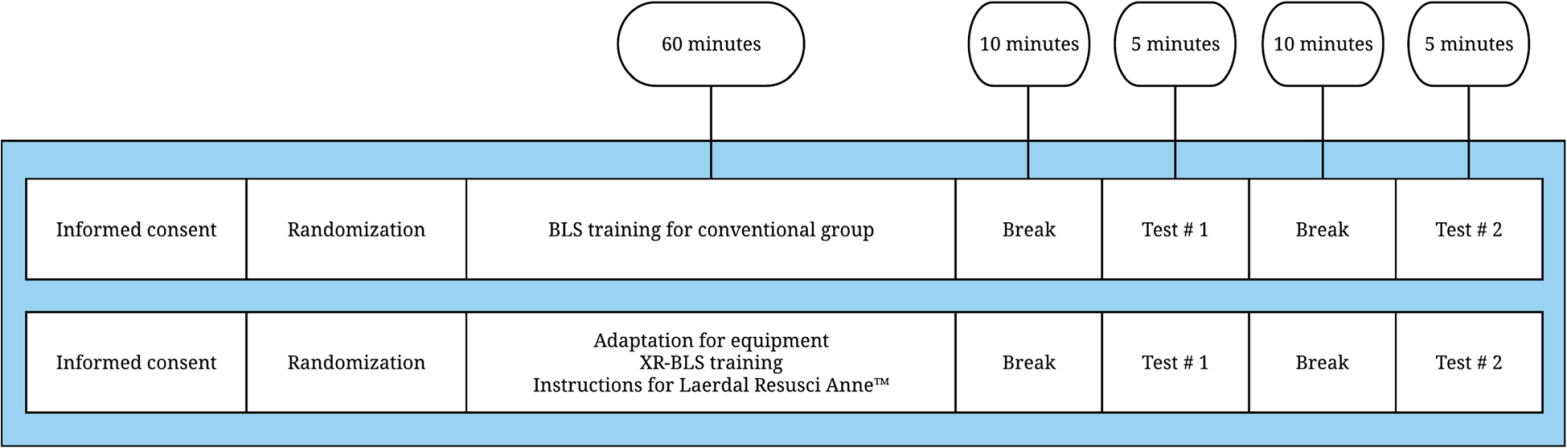
The participant timelines. Test #1, hands-only CPR; Test #2, 5 cycles of 30 compressions and a 10-s rest

The participant timelines are presented in Fig. 4.

### Sample size

This is a noninferiority clinical trial that compares the effectiveness of XR BLS training to that of conventional BLS training, which is based on a video. To calculate the sample size needed to assess the primary outcome of the mean number of chest compressions, which is determined in test #1, we defined a noninferiority margin on the basis of previous studies [7, 10, 11, 20, 21]. Since conventional training increased chest compression depth by 5 mm and a decrease in depth by 5 mm was reported to be associated with reduced survival after cardiac arrest [20, 21], the noninferiority margin was defined as − 5 mm. With an expected standard deviation of 10 mm [10, 11, 20], a one-sided alpha of 5%, and a power of 90%, each group needed 69 participants. After adjusting for a 10% drop-out rate, 77 participants are needed in each group.

### Recruitment

Volunteers will be recruited through announcements published on the official website of each institution as well as via direct contact or email invitation. Participants will be enrolled competitively, but a minimum of 32 participants will be enrolled from each participating institution.

Regarding enrolment and allocation, once the participant informs the researcher of his or her intention to participate in the study through the website or email or directly to the researcher, the date will be coordinated with the researcher, and then informed consent will be obtained. After that, randomisation will be conducted. If there are not enough participants, the period will be extended to encourage voluntary participation.

Since this study is for nonhealthcare providers, nonhealthcare providers from each institution can all be potential participants. In case of significant delays in enrolment, we will visit the department where nonhealthcare providers work to inform them of the study objectives and encourage participation.

Furthermore, the possibility of self-selection bias exists, which causes those interested in CPR to participate in the study. However, considering that the primary outcome of this study is a comparison of chest compression depth between the two educational methods, self-selection bias may not cause significant bias in the interpretation of the results.

Recruitment will be completed once the required number of volunteers has been obtained. There will be no officially paid incentives to participate in this study, but small gifts may be provided depending on the institutional conditions.

## Assignment of interventions: allocation

### Sequence generation

Participants will be enrolled competitively, but a minimum of 32 participants will be enrolled from each participating institution to minimise racial and physical differences among the participants in each country. Random numbers will be generated with the stratified randomised permuted block design generated in the SAS software (version 9.4; SAS Institute Inc., Cary, NC, USA) by an independent statistician, and the randomisation sequence will be implemented in the Research Electronic Data Capture (REDCap) software (version 9.5.2, Vanderbilt University, Nashville, TN, USA). Institution and sex will be applied as the stratification factors, and REDCap will randomly allocate participants to the XR or conventional group.

### Concealment mechanism

REDCap will use its algorithm for randomisation, which is unknown to the investigators.

### Implementation

Researchers from each institution will enrol participants, and randomisation sequences will be performed with REDCap. After randomisation, the researcher will send the participants to room A (conventional group) and room B (XR group) according to the results of randomisation. Participants will receive corresponding training in each room.

## Data collection and management

### Data management

The answers to the survey questions will be entered directly into REDCap or provided on paper and then entered into REDCap. REDCap is the software toolset and workflow methodology for electronic data collection and management. The person who will enter data for each institution has access only to their institution’s data. If the values that are entered are duplicated or out of range, the Trial Management Group (TMG) will confirm them (Tables 1, 2 and 3). REDCap servers are managed by Seoul National University Bundang Hospital. All web-based information transmission will be encrypted. All protocols, consent forms, and data will be stored in the REDCap system. The collected data will be stored for up to 6 months after the end of the study by the independent data coordinating centre. When it is necessary to store data for a longer period, the data could be sent to the research administration team to be stored for up to 3 years.

### Confidentiality

No personal identifiable information will be stored. Other data will be discarded after this study.

## Statistical methods

### Statistical analysis of the primary and secondary outcomes

Data will be analysed using SPSS 25.0 (IBM SPSS, Inc., Chicago, IL, USA) and Stata 10.0 (Stata Corp, College Station, TX, USA). The primary outcome will be assessed for noninferiority using a 1-sided 95% confidence interval of the mean compression depth between the XR and conventional groups. If the lower limit of the confidence interval is above the prespecified noninferiority limit of − 5, then the noninferiority of XR to conventional training will be established. Continuous variables will be presented as the means ± standard deviations (SDs) or medians with interquartile ranges (IQRs) after the normality of their distributions has been assessed with the Shapiro-Wilk test. The chi-square test or Fisher’s exact test will be used for comparisons of nominal variables, while the independent *t*-test and the Mann-Whitney *U* test will be used to compare continuous variables.

### Interim analyses

There are no planned interim analyses or subgroup or adjusted analyses in the trial.

### Methods for additional analyses

There is a plan to conduct a cost-benefit analysis. In addition, further analysis of the data generated in this trial could be performed in the future. This is stated in the informed consent.

## Oversight and monitoring

### Composition of the coordinating centre and trial steering committee

The Trial Steering Committee will consist of a team at Seoul National University Bundang Hospital and peer reviewers from Children’s Hospital Los Angeles, Royal Manchester Children’s Hospital, and National University of Singapore. TSC will constantly monitor patient safety and data integrity and provide recommendations for the discontinuation of any or all of the study or the modification of any aspect of the protocol. Their roles include the preparation of the protocol and any revisions, the application to obtain approval from the Research Ethics Committee, and the publication of study reports.

Data quality will be monitored by the TMG. It will consist of one investigator at each institution, including two statisticians at Seoul National University Bundang Hospital, who will not be involved in the actual simulation study. If the entered values are duplicated or are out of range, TMG will check and correct the values. Two independent statisticians will be able to access the entire trial dataset, and one investigator from each institution will have access to only their institution’s data.

### Composition of the data monitoring committee, its role, and reporting structure

This study does not need an independent Data Monitoring Committee. We have no plan to perform or publish any interim analyses.

### Adverse event reporting and harms

Any adverse events occurring in the study will be reported to TSC and the IRB. If there is an adverse event that prevents a participant from completing the study, the participant will be withdrawn, and the investigators will provide all necessary support and treatment.

Due to the nature of the simulation study, it does not cause significant harm to participants, and it was reported that VR did not cause significant harm to humans [22–25]. In addition, the study time is as short as 90 min in total. Therefore, no guidelines have been established for the termination of the trial.

### Plans for auditing trial conduct and audit frequency

Because this study is intended to be completed within 6 months, we do not have a plan for auditing the conduct of the trial.

### Plans for communicating important protocol amendments to relevant parties

If there are important protocol modifications, all investigators will be notified immediately via email.

### Dissemination plans

We will disseminate our findings through journal publications and conference presentations. If participants want to receive a report of the study results, we will send it via email after the study is complete.

## Discussion

In industrialised countries, OHCA is one of the most common causes of death; every year, 1 in 1000 people die from OHCA [15]. Despite many attempts to improve the survival rate, it is still between 6.7 and 8.4% [16].

According to the International Liaison Committee on Resuscitation (ILCOR), bystander CPR is associated with an improved survival rate and neurological recovery [17–19]. Previous research found that bystander CPR was more likely to be performed when laypersons had participated in CPR training [22]. Hence, increased layperson CPR training is related to an increase in the survival rate after OHCA, including better neurological outcomes [20, 21].

Conventional CPR training consists of checking for a response, calling for help, checking for breathing, performing chest compressions, performing rescue breathing, and using an AED. In conventional CPR training, the trainees watch the video and follow the instructions on the manikins [23], and BLS instructors guide trainees throughout the training process. Although this training method helped increase the bystander CPR rate, it has limitations due to its lack of realism and immersion. Moreover, the emergence of COVID-19 has necessitated training that is not performed face-to-face.

VR is one of the solutions that could overcome these limitations. It has been used in CPR training to maximise realism and immersion, which could improve the effectiveness of training. Nas et al. [2] reported that VR training resulted in a similar chest compression rate to conventional training. However, VR also has critical limitations. Procedures such as chest compressions, ventilations, and defibrillations cannot be implemented as in real environments in VR settings [1]. Although Nas et al. [2] used a pillow to practise chest compression in VR, it was soft enough to be compressed more easily than a manikin, which might result in a shallower compression depth in the VR training group than in the conventional group.

Recently, extended reality (XR), which combines the virtual and real worlds, was introduced. It could overcome the limitations of VR by embedding a real-world manikin into virtual environments, enabling trainees to actually practise chest compressions, rescue breaths, and defibrillation with the manikins.

The aim of this study was to compare the validity and efficacy of the XR-based BLS training method to those of the conventional training method. In the future, there will be major changes in the education field because of advanced technologies. Especially after the COVID-19 pandemic, the demand for remote education will increase, and CPR will be included in this trend. This study will be meaningful as a guide for future CPR training methods.

## Trial status

The recruitment of participants for this study will start in June 2021.

Recruitment is expected to be completed in December 2021.

Current protocol version and date: V1.0, 11 April 2021.

## Supplementary Information

**Additional file 1.** SPIRIT 2013 Checklist: Recommended items to address in a clinical trial protocol and related documents*.

**Additional file 2.** CBS 2.0 Survey (Conventional group).

**Additional file 3.** CBS 2.0 Survey (XR group).

**Additional file 4.** STUDY INFORMATION AND INFORMED CONSENT FORM.

**Additional file 5: Table S1.** Trial registration data.

**Additional file 6.** Supplementary video.

## Data Availability

After the completion of the study, the results will be disseminated through conferences and scientific publications in journals related to emergency medicine, resuscitation medicine, and medical education. In addition, all or part of the data without personal identifiable information will be available for online provision from the corresponding author upon reasonable request.

## Abbreviations

VR: Virtual reality
XR: Extended reality
CPR: Cardiopulmonary resuscitation
BLS: Basic life support
REDCap: Research electronic data capture
RCT: Randomised clinical trial
