# Supplementary material for: Comparison of extended reality and conventional methods of basic life support training: protocol for a multinational, pragmatic, noninferiority, randomised clinical trial (XR BLS trial)
Source: Trials. 2021 Dec 20;22:946. doi: 10.1186/s13063-021-05908-z (PMC8687636; doi:10.1186/s13063-021-05908-z)
Supplement: Supplementary file 2 — Additional file 2. CBS 2.0 Survey (Conventional group). [file 13063_2021_5908_MOESM2_ESM.docx]

**CBS 2.0 Survey (Conventional group)**

| **Participant number** |  | Date(DD/MM/YY) : |  |
| --- | --- | --- | --- |
| Tetrasignum Co., Ltd. specializes in developing medical educational content and related services using the latest digital technologies. Our first project CBS, a CPR education platform using VR, aims to inform people of the need for training and increase confidence in performing CPR in real life. Today’s experience is the first beta-test version, and we want to improve our services based on the information we obtain from this survey. Your response will be only used for content improvement, further research and development purposes. Your information will not be provided to any third parties.  Please try to give detailed answers. Thank you in advance for your precious time. | | | |

**1. Basic Information**

| 1. Gender | | | | | | | | | |
| --- | --- | --- | --- | --- | --- | --- | --- | --- | --- |
| ① Male | | | ② Female | | ③ Prefer not to say | | |  |  |
|  | | |  | |  | | |  |  |
| 2. Age  Please enter your age:  3. Height & Weight | | | | | | | | | |
| Height: | cm | OR | | feet/inches | Weight: | kg | OR | pounds |  |
| 4. Have you ever had CPR training before? | | | | | | | | | |
| 1. None (Go to 6) | | | ② 6mths ago | | ③ 6mths ~ 1yr ago | | | ④ 1 ~ 2yrs ago | ⑤ more than 2 yrs ago |
| 5-1. If you have, how many times have you received CPR training? | | | | | | | | | |
| ① Once | | | ② 2 times | | ③ more than 3 times | | |  |  |
| 5-2. If you have, where were you trained? (multiple choice) | | | | | | | | | |
| ① Primary school | | | ② Middle school | | ③ High school | | | ④ University or similar | ⑤ Military |
| ⑥ Work ⑦ Etc. ( ) | | | | | | | | | |
| 6-1. Have you ever used VR before? | | | | | | | | | |
| ① No (Go to 7) | | | ② Once | | ③ 2 times | | | ④ more than 3 times | ⑤ I have a VR device. |
| 6-2. If you have, what type of content have you experienced? | | | | | | | | | |
| ① Riding (Cars, Roller Coasters etc.) | | | ② Horror | | ③ Shooting games | | | ④ 360 Video | ⑤ Educational |
| ⑥ Other ( )  7. If you would like to receive reports on research results, please provide your email address.  ( ) | | | | | | | | | |

| Whether training is delivered well | | Strongly Agree | Agree | Neutral | Disagree | Strongly Disagree |  |
| --- | --- | --- | --- | --- | --- | --- | --- |
|  |  |  |  |  |  |  |  |
| 1 | During training, I felt as if I was performing CPR on an actual cardiac arrest patient. | 5 | 4 | 3 | 2 | 1 |  |
| 2 | I have received real time feedback on the depth and speed of chest compressions. | 5 | 4 | 3 | 2 | 1 |  |
| 3 | Through training, I have become proficient at performing CPR. | 5 | 4 | 3 | 2 | 1 |  |
| 4 | I believe that when I encounter suspected cardiac arrest patients, I can approach them without hesitation | 5 | 4 | 3 | 2 | 1 |  |
| 5 | I believe that when I witness a cardiac arrest patient, I can perform CPR confidently. | 5 | 4 | 3 | 2 | 1 |  |
