# Supplementary material for: Comparison of extended reality and conventional methods of basic life support training: protocol for a multinational, pragmatic, noninferiority, randomised clinical trial (XR BLS trial)
Source: Trials. 2021 Dec 20;22:946. doi: 10.1186/s13063-021-05908-z (PMC8687636; doi:10.1186/s13063-021-05908-z)
Supplement: Supplementary file 4 — Additional file 4. STUDY INFORMATION AND INFORMED CONSENT FORM. [file 13063_2021_5908_MOESM4_ESM.docx]

**STUDY INFORMATION
AND INFORMED CONSENT FORM**

Subject screening number: ________________

**Study Title: Comparison of extended reality and conventional methods of basic life support training: protocol for a multinational, pragmatic, non-inferiority, randomized clinical trial (XR BLS trial)**

**Chief Investigator:** Professor You Hwan Jo, Department of Emergency Medicine, Seoul National University Bundang Hospital

Dear Participant,

We would like to invite you to take part in our study. Before you decide to participate, it is important to understand exactly why we are conducting this study and how it will be conducted.

The following information is intended to explain the content of this study and your role in its participation and progress. Please take the time to read this participant manual and discuss it with your family members or other relevant individuals. In addition, if you have any questions, please ask the Chief Investigator or another Clinical Investigator. Be sure to think carefully before deciding whether or not to participate in this study.

1. **Purpose of the Study**

This clinical trial is being conducted for research purposes only.

**2. Background**

High-quality cardiopulmonary resuscitation is associated with good neurological outcomes from patients who have suffered from cardiac arrest. As such, proper training to ensure high-quality cardiopulmonary resuscitation plays an important role in the treatment of cardiac arrest patients.

The American Heart Association recommends that high-quality CPR consists of appropriate chest compression depth, between 5cm - 6cm, at a rate of 100-120 times per minute; with complete chest relaxation, minimal interruptions of chest compressions, without excessive ventilation, alternating chest compressors every two minutes, and CPR carried out at the ratio of 30 compressions to 2 rescue breaths.

This study will compare the effectiveness of existing CPR education methods to Extended Reality (XR) based CPR training that aims to maximize realism and immersion.

**3. Background information on medications/medical devices used for this clinical trial and the**

**probability of random assignment**

Not applicable to this clinical trial.

**4. Various tests or procedures that the subject will undergo in the clinical trial**

Individuals participating in the study will be randomly divided into two groups (a traditional CPR education group vs an extended reality CPR education group) and each group will receive CPR training in separate training spaces.

There are two evaluation processes in total (first evaluation: at the end of a practice session, second evaluation: after a 10-minute break), and both groups will undergo skill training that will include the steps: checking for response, asking for help, checking for breathing, chest compressions, rescue breaths and using an AED. The manikin to be used in the non-XR training group will be the Laerdal Resusci Anne™ (Laerdal Medical Corporation, Stavanger, Norway). CPR training and practice will last approximately 60 minutes.

**5. Conditions that the subject must comply with**

The Chief Investigator or other Clinical Investigators should be informed of any issues that might affect the participant’s performance (such as physical or cognitive limitations and including restrictions due to other factors e.g., age).

**6. The fact that it is an unproven clinical trial**

Not applicable.

**7. Possible side effects, risks or discomfort to the participant**

Extended Reality CPR training should have no side effects for most participants, but there may be mild cases in a small number people.

If you experience severe throbbing, dizziness, headaches, or physical pain during the training, you may be excluded or disallowed to continue from participating in the study.

**8. Expected benefits from the study**

You can improve your CPR skills and aid in furthering our research for the greater good.

**9. Alternative treatment (other alternative treatments other than clinical testing)**

Not applicable.

**10. Damage and Compensation**

It is very unlikely that there will be possible side effects from this study.

If side effects do occur, researchers will do their best to provide appropriate treatment.

**11. Financial Compensation**

Not applicable.

**12. Estimated Costs**

Not applicable.

**13. Voluntary participation**

This is a written explanation and consent form presented to all research participants.

This is a study meant to show the effectiveness of education, and the study participants may refuse to participate in the test or decline to continue at any time during the trial.

**14. Personal information**

In order to verify that the quality of the clinical trial and data collection is in accordance with the relevant laws, the monitoring personnel, the review committee, and the head of the Ministry of Food and Drug Safety Association will be allowed to access the personal information of the participant, upon request.

**15. Securing confidentiality**

All data shall be recorded and managed by the appointed research manager, and the collected data shall not be available to anyone other than authorized personnel.

The data collected for each subject will be collected as evidence for our records, and the collected data will be stored in a password protected Excel file.

There could be further analyses of the data generated in this trial.

Research records will be kept for three years, starting from the end of the study, and will be destroyed by the storage institution upon reaching the 3-year mark.

Records that contain the individual's identity will be kept confidential and will remain so if the results of the clinical trial are published.

The individual’s personal information that is obtained during the study will not be provided to any third parties.

**16. The continuous provision of new research information**

We will notify the participant or a representative in a timely manner if we acquire new information that could affect the participant's continued participation in the clinical trial.

**17. Contact us**

In the event of any issues, concerns, or questions arising from our clinical research:

Please contact Seul-ki Choi at Seoul National University Bundang Hospital (031-787-3031)

or

You can contact the IRB or Clinical Research Ethics Center to consult with any questions, concerns, or concerns about the rights and interests of the research (031-787-8801~5)

**18. Discontinuation of the study**

Discontinuation of the study for individual participants will occur in the following cases: where individuals express an intention to drop out of the study during the trial or data collected cannot be analyzed due to system error.

**19. Participant time requirement for the clinical trial**

The expected participation period for the clinical trial is 1 day, and if further information is needed, you will be contacted by phone or email.

**20. Approximate number of individuals participating in the clinical trials**

Target number of participants: 154 people (a minimum of 32 participants from each country, selected through competitive recruitment).

**21. Other**

If you are interested in participating after hearing the details of this study, we would like you to sign a separate consent form.

If the participant or their proxy is unable to read the consent form, instructions, or other documented information, the attending researcher must be present when consent is being obtained.

Records of personal information that can identify the subject's identity will be kept confidential. Monitoring personnel of clinical research, inspectors, IRB and the Minister of Health and Welfare can access the subject's research records to the extent that they are protected by relevant laws.

We will not provide personal information to third parties not involved in this study for secondary use.

You will be provided instructions as explained and one copy of the signed consent form.

**Consent of Research Participants**

**Study Title: Basic Life Support training comparison between conventional and eXtended Reality methods: A parallel-group, multinational, randomized controlled trial (BLS XR trial)**

1. I have heard and fully understood all the information about this study from the researcher in detail.

2. I have also read the subject consent manual and understand that this study is being conducted for research purposes.

3. I understand that my decision to participate in the study is voluntary and that I can refuse or freely discontinue participation for personal reasons at any time during the study period, which will not result in any medical or other disadvantages.

4. I know that if side effects occur during the research process, the 'compensation manager' will be responsible according to the provisions on compensation for damages.

5. I know that if I have any questions about this study, I am able to contact the researcher at any time and I agree that researchers may read my personal information for the sole purposes of this study.

Therefore, I agree to participate in this study according to my free will.

|  | **Name** | **Name** | **Date of Signature** |
| --- | --- | --- | --- |
| **Participant** |  |  | Y/M/D |
| Participant’s Proxy  (If applicable) |  |  | Y/M/D |
|  | Relationship to the Participant: ___________________________________  Specifics: ____________________________________________________ | | |
| Clinical Investigator  (or co-researcher) |  |  | Y/M/D |

If applicable,

|  | **Name** | **Name** | **Date of Signature** |
| --- | --- | --- | --- |
| **Observer** |  |  | Y/M/D |
