# Supplementary material for: Comparison of extended reality and conventional methods of basic life support training: protocol for a multinational, pragmatic, noninferiority, randomised clinical trial (XR BLS trial)
Source: Trials. 2021 Dec 20;22:946. doi: 10.1186/s13063-021-05908-z (PMC8687636; doi:10.1186/s13063-021-05908-z)
Supplement: Supplementary file 5 — Additional file 5: Table S1. Trial registration data. [file 13063_2021_5908_MOESM5_ESM.docx]

Supplementary table 1. trial registration data

| Data category | Information |
| --- | --- |
| Primary registry and trial identifying number | ClinicalTrials.gov  NCT04736888 |
| Date of registration in primary registry | 29 January, 2021 |
| Source(s) of monetary or material support | National Research Foundation of Korea (NRF) grant funded by the Korean government (MSIT) and Seoul National University Bundang Hospital Research Fund |
| Primary sponsor | National Research Foundation of Korea (NRF) grant funded by the Korean government (MSIT) |
| Secondary sponsor(s) | Seoul National University Bundang Hospital Research Fund |
| Contact for public queries | Chang Woo Im, MD [goalforall@snubh.org] |
| Contact for scientific queries | Chang Woo Im, MD  Seoul National University Bundang Hospital |
| Public title | Comparison of extended reality and conventional methods of basic life support training: protocol for a multinational pragmatic clinical trial (XR BLS trial) |
| Scientific title | Comparison of extended reality and conventional methods of basic life support training: protocol for a multinational pragmatic clinical trial (XR BLS trial) |
| Countries of recruitment | Republic of Korea, The United States of America, Singapore, the United Kingdom (UK) |
| Health condition(s) or problem(s) studied | CPR training using Extended reality |
| Intervention(s) | Conventional group: Conventional CPR training |
|  | XR group: CPR training with XR module |
| Key inclusion and exclusion criteria | Ages eligible for study: ≥18 years  Sexes eligible for study: Both  Accepts Healthy Volunteers: Yes |
|  | Inclusion criteria: Individuals who are not healthcare providers and are 18 years old or olde |
|  | Exclusion criteria:  Participants who   - are not capable of performing either the training or the CPR test due to physical or cognitive limitations - have upper extremity injuries or are pregnant - experience dizziness, headache, or motion sickness during the 2-minute XR device adaptation period that prevents them from participating in the simulation study |
| Study type | Interventional |
|  | Allocation: multinational, pragmatic, non-inferiority, randomized clinical trial |
|  | Primary purpose: To compare BLS skills gained by the XR method to those gained by the conventional method |
| Date of first enrolment | September 2021 |
| Target sample size | 154 |
| Recruitment status | Recruiting |
| Primary outcome(s) | The mean compression depth (mm) over 2 minutes |
| Key secondary outcomes | The total number of chest compressions (n)  The mean chest compression depth of each 30-second epoch  Correct hand position (%)  Adequate compression depth (%)  Compression and full release (%)  Mean compression rate (number per minute) Adequate compression rate (%)  Adequate compression depth and rate (%)  Hands-off time (sec). |
